# Physical activity before and during the COVID-19 pandemic in Vojvodina, Serbia

**DOI:** 10.3389/fpubh.2022.993035

**Published:** 2022-09-27

**Authors:** Jelena Obradović, Nikola Radulović, Duško Cvijović, Mila Vukadinović Jurišić

**Affiliations:** Faculty of Sport and Physical Education, University of Novi Sad, Novi Sad, Serbia

**Keywords:** coronavirus disease, the activity of the adult population, IPAQ-SF, physical activity, public health

## Abstract

**Background:**

The physical activity (PA) of the youth and adult population underwent changes during the last 2 years due to the coronavirus (COVID-19) pandemic, and all for the purpose of maintaining the health of the population. The purpose of this study was to estimate the levels of PA in the youth population and the adult population (young and old) during the COVID-19 pandemic in the territory of Vojvodina, Serbia, and to determine the differences between them, as well as to compare these results with the results before the pandemic.

**Methods:**

A total of 1,117 subjects (age 36.27 ± 15.08 years) from the territory of Vojvodina, Serbia, participated in the study. Subjects were recruited and assigned to one of the three groups according to their age: youth group (*N* = 395; age 18–24 years), young adults group (*N* = 347; from 25 to 44 years), and old adults group (*N* = 375; age 45–65 years). All participants in this study completed the International Physical Activity Questionnaire Short-Form between July and November 2021, during the fourth wave of the COVID-19 pandemic in Vojvodina, Serbia. The Kruskal–Wallis test and Dunn multiple comparison *post-hoc* method were used for statistical analyses.

**Results:**

The youth showed the highest result in total weekly energy expenditure (3,893.72 ± 2,010.01 MET-min/week) compared to young adults (2,528.20 ± 1,804.11 MET-min/week) and old adults (2,369.07 ± 2,084.95 MET-min/week) during the COVID-19 pandemic in Vojvodina, Serbia. In addition, adults (young and old) spent more time sitting than youth during the same period. Furthermore, the youth achieved greater results in levels of PA during the COVID-19 pandemic compared to the situation before the COVID-19 pandemic. However, adult populations achieved lower results during the COVID-19 pandemic than before the COVID-19 pandemic.

**Conclusion:**

During the COVID-19 pandemic, the youth from Vojovida, Serbia, achieved greater results in PA levels than the adult populations. Based on that, we recommend that it is necessary to take steps toward increasing PA in the adult population, especially old adults.

## Introduction

The respiratory syndrome coronavirus disease 2019 (COVID-19) was first detected in Wuhan, Hubei Province, China, in December 2019, and the spread of the virus continued beyond the borders of China. Older adults were at the highest risk of infection from the coronavirus (1, 2), but youth and young adults were also endangered (3). Due to the danger to public health of international importance, the World Health Organization declared a pandemic on 11 March 2020 (4). The first case of COVID-19 in the Republic of Serbia was registered on 6 March 2020, and increased very rapidly. The Government of the Republic of Serbia declared a pandemic on 20 March 2020, when a state of emergency and a curfew were introduced. Furthermore, 17 European countries also declared the pandemic, and thus they started the fight against the virus with the introduction of restrictive measures (5). The strategies applied by the states at the beginning of the pandemic differed, from a very liberal approach to the introduction of extremely restrictive measures, all with the aim of preserving the health of the population. Therefore, quarantine combined with other restrictive measures can reduce transmission of the virus (6) but increase physical inactivity (PI) (7–9).

According to the data of the World Health Report (10), the PI represents a global problem from childhood to old age before the pandemic. A detailed statistical analysis shows that 31.1% of the adult population is physically inactive worldwide. Furthermore, during the COVID-19 pandemic, the PI increased further, which is confirmed by previous reports (11–14). The study (11) reported that during the COVID-19 pandemic in nine European countries (Bosnia and Herzegovina, Croatia, Greece, Kosovo^*^, Italy, Slovakia, Slovenia, Spain, and Serbia), the PI and screen time in the adult population increased. Furthermore, in Croatia (12) and Spain (13, 14), during the COVID-19 pandemic (from January to April 2020), the PI increased in adolescents. In Canada and Brazil, the COVID-19 pandemic (between April and May 2020) negatively affected physical activity (PA) in young adults (15) and old adults (16). In Qatar, Hermassi et al. (17) reported that the COVID-19 pandemic reduced vigorous-intensity PA from 663 ± 320 metabolic equivalents (MET-min/week) to 323 ± 187 MET-min/week in young adults. Accordingly, it can be observed that the adult population before the COVID-19 pandemic (10) and during the COVID-19 pandemic (11, 15–17) is physically inactive. This is concerning because PI is one of the leading modifiable risk factors for global mortality (18). In Vojvodina, Serbia, the leading causes of mortality in the population are cardiovascular diseases (CVDs) (56.8%) and cancer (19.7%) (19). Therefore, it is important that the population of Vojvodina, Serbia, be physically active because the PA effectively protects from cardiovascular diseases and cancer (20), and thus mortality of the population. To the authors' knowledge, no studies were found that assessment and analyzed of PA population with the majority of CVD cases in Europe (21) has never been investigated. Also, no study assessed the PA in the population 2 years after the declared COVID-19 pandemic; these gaps are addressed in this study. Therefore, the evaluation of the PA in the population, as well as the recommendations for improving PA, is important because excessive mortality and death are related to diseases that are often caused by a decrease in PA. Based on these findings, the purpose of this study was to estimate the levels of PA in both the youth population and the adult population (young and old) during the COVID-19 pandemic in the territory of Vojvodina, Serbia, and to determine the differences between them, as well as to compare these results with the results before the pandemic.

## Materials and methods

### Data collection

All participants completed the online questionnaire during the COVID-19 pandemic (fourth wave) in the territory of Vojvodina, Republic of Serbia, between 8 July 2021 and 3 November 2021. This questionnaire consisted of two sets of questions. One set consisted of questions about sociodemographic factors, and the other set included questions from the International Physical Activity Questionnaire Short-Form (IPAQ-SF). Several studies argue that IPAQ-SF has high reliability and validity for measuring PA in the adult population (22–24). Therefore, the authors (25, 26) applied this questionnaire to measure PA in the youth and adult population before the pandemic and during the COVID-19 pandemic (27–30). The other questionnaires for measuring PA during the COVID-19 pandemic were also applied, such as the Godin Leisure Questionnaire (15) and the Spanish short version of the Minnesota Leisure-Time Physical Activity Questionnaire (30). Based on the results, it can be concluded that IPAQ-SF should not be the only questionnaire for measuring PA during the COVID-19 pandemic, but it is the most applicable of all the questionnaires. In addition, the evaluation of PA and the results obtained can be compared with similar studies performed in our country and other countries during the COVID-19 pandemic. The IPAQ-SF was taken from the official website of the International Physical Activity Questionnaire (31) and was translated into the Serbian language by two independent experts who were familiar with this type of questionnaire. The accuracy of the questionnaire was checked, and certain differences in translation were removed. The online questionnaire was made in electronic form (Google questionnaire). To survey a sample of respondents as large as possible, one version of the questionnaire was made in electronic form (Google questionnaire) and was shared *via* e-mail, Viber™, and WhatsApp™. Each participant completed the questionnaire only once, and responses were anonymous and confidential according to Google's privacy policy.

### Subjects

All 1, 117 participants (male: *N* = 558; age 33.53 ± 15.43 years and female: *N* = 559: age 39.01 ± 14.21 years) from the territory of Vojvodina, Republic of Serbia, during the COVID-19 pandemic (fourth wave) participated in the study. **I**nclusion criteria were as follows: (i) individuals living in the territory of Vojvodina, Serbia; (ii) age ≥ 18 years; and (iii) all individuals who voluntarily agreed to participate in the study. Considering that the territory of the Vojvodina is a multi-ethnic environment, there were no restrictions on nationality. Furthermore, there were no restrictions on gender, occupation, or socioeconomic level of the participants. They were no specific exclusion criteria. The study included all participants who met the inclusion criteria and divided them into three age groups. According to Celis-Morales et al. (32), age classifications were divided into three categories: youth (from 15 to 24 years), young adults (from 25 to 44 years), and old adults (from 45 to 65 years). Table 1 shows the sociodemographic characteristics of all participants. All participants signed an informed consent (which was at the beginning of the questionnaire) form to participate in the study. The Ethics Committee of the Faculty of Sport and Physical Education, the University of Novi Sad, approved the study (No-47-10-09/2021-1). All procedures were conducted according to the Declaration of Helsinki.

**Table 1:** Sociodemographic characteristics of all participants according to age categories.

**Group**		**Youth**	**Young adults**	**Old adults**
*n*		395	347	375
Years (Mean ± SD)		19.89 ± 1.44	35.83 ± 5.67	53.95 ± 7.04
Male *n* (%)		261 (66.1%)	142 (40.9%)	155 (41.3%)
Female *n* (%)		134 (33.9%)	205 (59.1%)	220 (58.7%)
Environment	Village (%)	13.2	10.4	17
	Small town (%)	47.1	41.2	43.5
	Big city (%)	39.7	48.4	48.4
	Elementary (%)	62.8	0.3	2.9
Education	High school (%)	37.2	29.4	47.2
	Faculty (%)	0	70.3	49.9

During this period (the fourth wave of the COVID-19 pandemic), the Government of the Republic of Serbia introduced preventive measures, including teaching at the faculties organized in the combination methods (direct teaching or e-learning at home); training centers were opened, but with special restrictions (athletes were obliged to wear protective masks and respect the 2-m physical distance in all enclosed spaces); vaccination of the population was underway; mandatory covid passes were introduced; a limit of 500 people indoors was introduced; employees in companies were working at home; and elderly citizens of Serbia were allowed to move at certain hours, with the recommendation to be vaccinated (33).

### The online questionnaire

The online questionnaire was made in electronic form (Google questionnaire) and consisted of 2 sets of questions, where the first set included four questions about sociodemographic factors, such as “Gender,” “Years,” “Education,” and “Environment in which people live.” The other set of questions was taken from IPAQ-SF and records the activity of four intensity levels: vigorous-intensity PA (VPA: doing heavy lifting, performing intense aerobic exercises, and using a bike or treadmill); moderate-intensity PA (MPA: carrying light loads and cycling at a regular pace, and working out in the yard); and walking time (W), as well as the average time spent sitting (ST) on a weekday, including sitting at work, in the last 7 days. Also, this questionnaire provides information on PA level as energy expenditure in MET-min/week.

The authors of this study calculated the weekly PA levels (VPA, MPA, and W) expressed in MET-min/week. For each type of PA, MET-min/week coefficients were calculated through Microsoft Excel spreadsheet automatic scoring of the IPAQ-SF, according to Cheng (34) with the following results: 3.3 for W, 4.0 for MPA, and 8.0 for VPA. Also, we estimated the total weekly energy expenditure (Total PA), which is the sum of W, MPA, and VPA in MET-min/week (34).

### Statistical analyses

The minimum sample size was calculated using Minitab Statistical Software Version 18 by using the results obtained from a similar study. The total sample size of the study was calculated to be at least 987 participants; a significant difference (*p* = 0.05) for the type I error was set at 5% and the power of the study was set at 80%. The total number of individuals who participated in this study was 1,117. The other statistical analysis was performed with the SPSS statistical program version 20 (SPSS Inc., Chicago, IL, USA). The results are presented as mean ± standard deviation (SD). The Kolmogorov–Smirnov test was used to determine normality distribution for all variables across the age. The Kruskal–Walis test was used to compare the results across all three groups for each level of PA. In addition, Dunn *post-hoc* test was used to determine which pairs of variables showed significant differences. The statistical significance was set at *p* ≤ 0.001.

## Results

All 1,117 participants (males, *N* = 558: age 33.53 ± 15.43 years and females, *N* = 559: age 39.01 ± 14.21 years) were classified into three groups according to their age. Table 1 presents the sociodemographic characteristics of all participants according to their age during the COVID-19 pandemic in Vojvodina, Serbia.

Table 2 shows the descriptive statistics of youth, young, and old adults in VPA (days per week and min per week), MPA (days per week and min per week), W (days per week and min per week), Total PA (days per week), and ST (hours per week) during the COVID-19 pandemic in Vojvodina, Serbia.

**Table 2:** Descriptive statistics of youth, young adults, and old adults in levels of PA during the COVID-19 pandemic.

	**Group**	**Mean rank**	**Chi-square**	***p***
VPA (MET-min/week)	Youth	713.37	151.39	0.000*
	Young adults	503.90		
	Old adults	447.38		
MPA (MET-min/week)	Youth	633.00	32.70	0.000*
	Young adults	513.32		
	Old adults	523.32		
W (MET-min/week)	Youth	618.60	25.07	0.000*
	Young adults	551.25		
	Old adults	503.39		
Total PA (MET-min/week)	Youth	708.77	136.15	0.000*
	Young adults	503.34		
	Old adults	452.75		
ST (h)	Youth	456.87	60.54	0.000*
	Young adults	604.27		
	Old adults	619.42		

VPA, vigorous-intensity PA; MPA, moderate-intensity PA; W, walking time; Total PA, Total weekly energy expenditure of PA; ST, sitting time.

In Table 3, the results of the Kruskal–Wallis test show a significant difference (*p* ≤ 0.001) at all the levels of PA between the groups.

**Table 3:** Results of Kruskal–Wallis test in levels of PA for youth and adult population during the COVID-19 pandemic.

**Variables**	**Units**	**Youth**	**Young adults**	**Old adults**
		**(Mean ± SD)**	**(Mean ± SD)**	**(Mean ± SD)**
VPA	Days per week	3.31 ± 1.98	1.90 ± 1.82	1.58 ± 1.89
	Min per week	66.37 ± 27.80	48.89 ± 32.57	55.66 ± 34.25
MPA	Days per week	3.52 ± 2.01	2.65 ± 2.00	2.89 ± 2.24
	Min per week	61.28 ± 29.76	50.10 ± 30.98	52.39 ± 30.57
W	Days per week	6.01 ± 1.65	5.27 ± 2.06	4.87 ± 2.15
	Min per week	59.82 ± 31.17	55.20 ± 29.94	54.34 ± 28.55
Total PA	Days per week	6.38 ± 1.47	4.93 ± 3.55	4.91 ± 2.64
ST	Hours per week	3.51 ± 1.95	4.93 ± 3.55	4.91 ± 2.64

VPA, vigorous-intensity PA; MPA, moderate-intensity PA; W, walking time; Total PA, Total weekly energy expenditure of PA; ST, sitting time;

* statistically significant differences between the groups (*p* ≤ 0.001).

**Figure 1:**
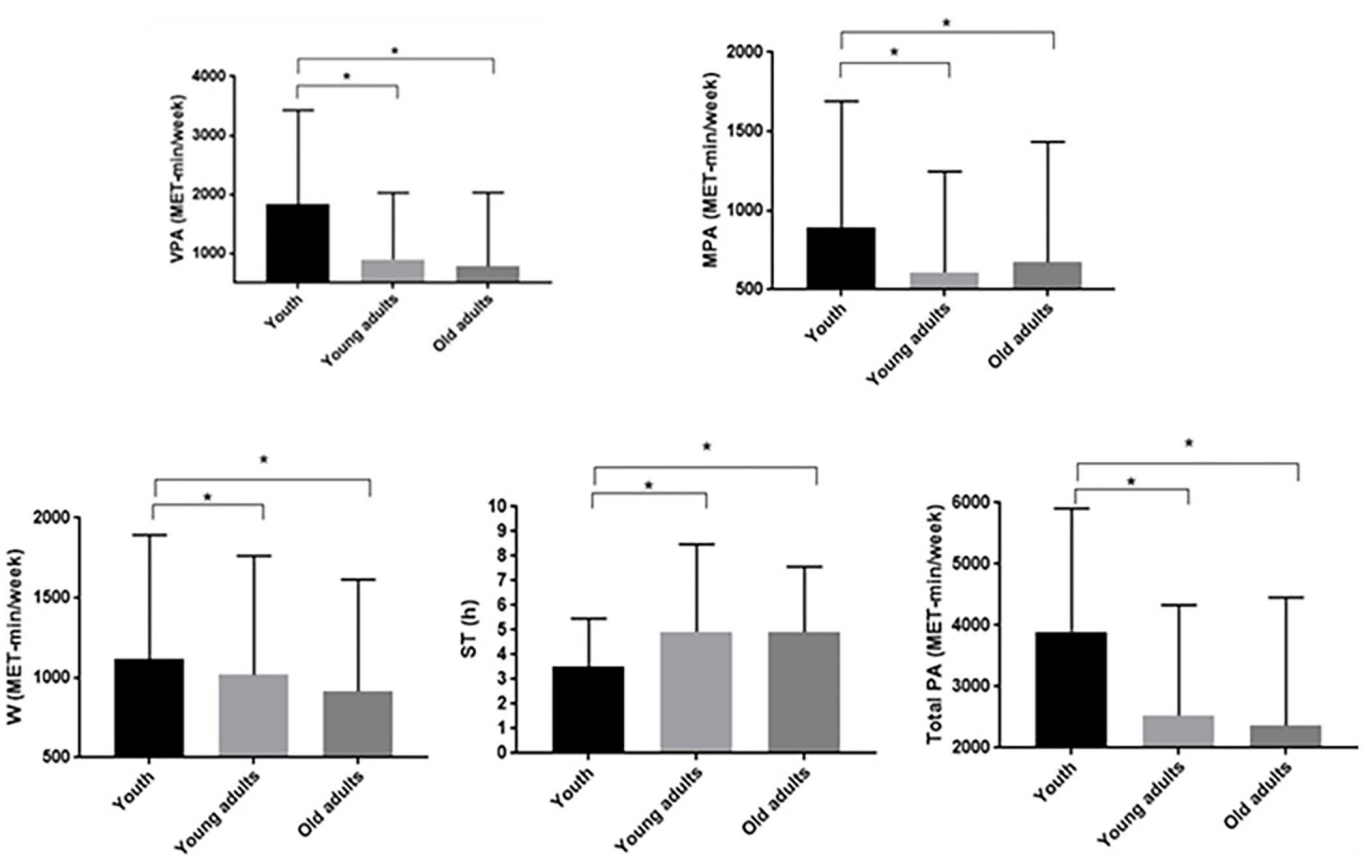
The levels of PA between the groups during the COVID-19 pandemic. VPA, vigorous-intensity PA; MPA, moderate-intensity PA; W, walking time; ST, sitting time; Total PA, Total weekly energy expenditure of PA; *statistically significant differences between the groups (*p* ≤ 0.001).

The levels of PA between the groups during the COVID-19 pandemic are presented in Figure 1.

The youth participants spend more time in VPA than young and old adults (1,836.52 ± 1,592.43 MET-min/week vs. 900.22 ± 1,126.54 MET-min/week vs. 781.81 ± 1,251.96 MET-min/week) during the COVID-19 pandemic (*p* ≤ 0.001). Furthermore, youth participants achieved statistically significantly greater (p ≤ 0.001) results in MPA (890.48 ± 801.62 MET-min/week) than young adults (609.19 ± 637.79 MET-min/week) and old adults (673.26 ± 760.70 MET-min/week) during the COVID-19 pandemic in Vojvodina, Serbia. In addition, the youth participants reported the highest result in W during the COVID-19 pandemic in Vojvodina, Serbia (1,116.48 ± 776.97 MET-min/week) compared to the young adults (1,018.55 ± 743.64 MET-min/week) and old adults (913.79 ± 701.20 MET-min/week).

The old adults during the COVID-19 pandemic in Vojvodina, Serbia, spent more time sitting (4.91 ± 2.65 h) compared to the youth (3.51 ± 1.95 h). In addition, young adults (4.93 ± 3.55 h) spent more time sitting than the youth (3.51 ± 1.95 h).

The results presented in Figure 1 also show statistically significant differences (*p* < 0.001) between groups in Total PA (MET-min/week). Participants from the youth group achieved greater results (3,893.72 ± 2,010.01 MET-min/weeks) compared to other groups (young adults: 2,528.20 ± 1,804.11 MET-min/week and old adults: 2,434.48 ± 2,077.83) during the COVID-19 pandemic in Vojvodina, Serbia.

Table 4 shows the differences in the levels of PA before the COVID-19 pandemic (35) and during the COVID-19 pandemic in Vojvodina, Serbia. The youth population achieved greater results in the levels of PA during the COVID-19 pandemic compared to the situation before the COVID-19 pandemic. However, adult populations (young and old) achieved lower results during the COVID-19 pandemic than before the COVID-19 pandemic.

**Table 4:** Physical activity before and during the COVID-19 pandemic in Vojvodina, Serbia.

**Variables**	**Units**	**Youth**		**Young adults**		**Old adults**	
		**Before**	**During**	**Before**	**Duringc**	**Before**	**During**
		**pandemic**	**pandemic**	**pandemic**	**pandemic**	**pandemic**	**pandemic**
VPA	Days per week	2*	3.31	2*	1.90	1.85*	1.58
MPA	Days per week	2.5*	3.52	2.7*	2.67	3*	2.89
W	Days per week	5.7*	6.01	5*	5.27	4.85*	4.87
ST	Hours per week	from 5 to 8*	3.51	2.5*	4.93	from 2 to 5*	4.91

VPA, vigorous-intensity PA; MPA, moderate-intensity PA; W, walking time; Total PA, Total weekly energy expenditure of PA; ST, sitting time;

* Results before pandemic according to Eurobarometer 2020 (35).

## Discussion

The present study examined the levels of PA in both the youth population and the adult population (young and old) during the COVID-19 pandemic in the territory of Vojvodina, Serbia, and determined the differences between them. In addition, the results obtained before the pandemic and during the pandemic were compared. The major findings were that the youth population accomplished a greater result in Total PA (3,893.72 MET-min/week) in comparison to young adults (2,528.20 MET-min/week) and old adults (2,369.07 MET-min/week) during the COVID-19 pandemic in the territory of Vojvodina, Serbia. In addition, old adults spent more time sitting (4.91 ± 2.65 h) compared to youth (3.51 ± 1.95 h) and young adults (4.93 ± 3.55 h). Furthermore, the youth population achieved higher results in the levels of PA during the COVID-19 pandemic compared to the situation before the COVID-19 pandemic, but the adult population achieved lower results than before the COVID-19 pandemic. The youth population during the COVID-19 pandemic in Vojvodina, Serbia, achieved a greater result in Total PA (3,893.72 ± 2,010.01 MET-min/week) compared to young adults (2,528.20 ± 1,804.11 MET-min/week) and old adults (2,434.48 ± 2,077.83 MET-min/week). Magueri et al. (28) classified PA based on weekly MET achieved, where the low active group had less than 600 MET-min/week, the moderately active group had from 600 to 3,000 MET-min/week, and the physically active group achieved more than 3,000 MET-min/week. Our youth participants achieved more than 3,000 MET-min/week, which classified them as a physically active group, despite the peak of the fourth wave of the COVID-19 pandemic in Vojvodina, Serbia. However, in Italy, during the COVID-19 pandemic (from 1 April to 30 April 2020), the youth population achieved only 1,852 MET-min/week in Total PA (28). It is also important to note that the youth included in this study were students at the Faculty of Sport and Physical Education. Students in our sample were athletes previously, and hence they were expected to have higher levels of PA when compared to the general population (14). Also, they attended practical lectures at the faculty meeting during this research, which can also influence their PA levels. Additionally, it is assumed that restrictive measures (e.g., closing the schools and public places, and people could move only for essential activities) restricted the movement of the youth population, while in this study, it was not the case. In Vojvodina, Serbia, during this period of the COVID-19 pandemic, the number of infections rapidly increased from 74 (2 July 2021) to 6,948 (27 October 2021) (33). Therefore, the Government of the Republic of Serbia introduced restrictive measures (the school was organized by using combined methods, exercisers were using a protective mask, 2-m physical distance was maintained in all enclosed spaces, vaccination of the population was underway, mandatory COVID passes, and up to 500 people were allowed in indoors) but did not influence the movement of youth from Vojvodina, Serbia. When comparing results between the youth and adult populations at the levels of PA, we can observe that the youth population achieved higher results in VPA, MPA, and W than in young and old adults during the COVID-19 pandemic in Vojvodina, Serbia. Similar results were obtained in Italy during the COVID-19 pandemic, where youth and young adults reported higher levels of PA compared to old adults (27). Furthermore, Hallal et al. (36) demonstrated that young people were more physically active than old people before the COVID-19 pandemic. The differences between the youth, young adults, and old adult populations in the levels of PA before the COVID-19 pandemic were mostly due to the aging process (37). Additionally, the differences between youth and adult populations (young and old) in the levels of PA during the COVID-19 pandemic are also assumed to be a consequence of aging, but not the COVID-19 pandemic. During the fourth wave of the COVID-19 pandemic in Vojvodina, Serbia, the restrictive measures that were in force allowed all participants to exercise, but did not restrict their movement.

When interpreting the results according to Eurobarometer 2020 (35), the youth population before the COVID-19 pandemic achieved lower results in VPA compared to the youth population during the COVID-19 pandemic (2 days per week vs. 3.31 days per week). Furthermore, the youth population from Vojvodina, Serbia, during 2019 (before the pandemic) engaged in MPA for only 2.5 days per week (35), while the youth population from this study engaged in MPA for 3.52 days per week. Based on the average number of days in VPA and MPA, it can be concluded that the youth population spent more time in vigorous and moderate activities during the COVID-19 pandemic than before. However, it is important to note that youth from Eurobarometer (35) and youth from this study are from Vojvodina, Serbia, but with different occupations. The youth from our study are students from the Faculty of Sport and Physical Education, and they are commonly more active than other youth populations (14).

According to Eurobarometer 2020 (35), the young adults before the COVID-19 pandemic spent a longer time in vigorous and moderate PA than during the COVID-19 pandemic. In addition, young adults before the pandemic spent a shorter time sitting (2.5 h) when compared to young adults during the COVID-19 pandemic (4.93 h). Sedentary behavior is a characteristic of people in certain sociodemographic groups like citizens who stayed for longer in education, office staff, managers, and people who live in towns (35). Results from this study (see detailed Table 1) showed that 70.3% of young adults finished faculty and 48.4% lived in big cities, which indicates that they are employed as office workers and that sedentary behavior is characteristic of them. Also, further information about restrictive measures that were in force in 2021 in Serbia (AP Vojvodina), as well as excessive screen-based activities during the COVID-19 pandemic (38), confirmed these findings.

The old adults during the COVID-19 pandemic in Vojvodina, Serbia, accomplished lower results in VPA, MPA, and W compared to old adults before the pandemic in Vojvodina (35). It is well-known that PA can contribute to the physical and psychological well-being of old people (39–41). However, our results showed that the COVID-19 pandemic had a negative impact on PA, and thus on the health of old adults of Vojvodina, Serbia.

The strength of this study is a large sample of participants, and we used IPAQ-SF as the most useful questionnaire for estimating PA in youth and adults during the COVID-19 pandemic (42). Therefore, obtained results can be compared with the results of our study and other studies. There are a few limitations to this study. First, the PA levels were estimated for different ages, excluding the variables, such as gender, education level, and the environment in which the participants live. However, these variables could be taken into account in future studies. Second, no cookie-based protection was used to exclude the possibility of duplicates. Third, Lee et al. (24) note a low correlation with objective measures in the IPAQ-SF. Future research should continue to monitor the PA of the population after the pandemic, in order to prevent a pandemic of a sedentary lifestyle. Authors (43) have reported that PI is the fourth leading cause of mortality worldwide, and 31.1% of the adult population is physically inactive (36).

## Conclusion

In conclusion, the present study showed very large changes in all levels of PA after 2 years of the declared COVID-19 pandemic in the youth and adult population of Vojvodina, Serbia. However, COVID-19 infection has a greater influence on young adults and old adults than on the youth population. Therefore, our article is extremely important because this is the first study to analyze the levels of PA in the population of Vojvodina, Serbia. Also, this is the first study to analyze the levels of PA 2 years after the declaration of the COVID-19 pandemic, while previous studies (44–46) analyzed the levels of PA at the beginning of the COVID-19 pandemic. COVID-19 infection has a greater influence on adults than on the youth population of Vojvodina, Serbia. These findings provided clear evidence that adults from Vojvodina, Serbia, should be more aware of PA during the COVID-19 pandemic because of the big risk associated with low PA. This is important because the Serbian population leads to the number of CVDs in Europe (21). The occurrence of CVD is highly correlated to PI. Therefore, PI must not become a habit of the population of the territory of Vojvodina, Serbia. Furthermore, it is necessary to take steps toward increasing PA in the adult population, especially old adults from Vojvodina, Serbia. We propose several recommendations and practical implications to increase the PA of old adults, as they constitute one of the most endangered populations from the territory of Vojvodina during the COVID-19 pandemic:

- To increase public awareness about the importance and benefits of the PA through educational interventions.
- To promote PA that can be performed outside (using outdoor fitness gyms, riding bicycles, walking in nature, and getting involved in gardening).

To conclude, the mentioned recommendations can be extended to the general public, if the restrictive measures in the country allow them. The general public should be informed about the benefits of PA, and we should not allow PI (reduced PA due to the COVID-19 pandemic) to become a habit of the population.

## Data availability statement

The raw data supporting the conclusions of this article will be made available by the authors, without undue reservation.

## Ethics statement

The studies involving human participants were reviewed and approved by the study was conducted in accordance with the Declaration of Helsinki and approved by the Ethics Committee Faculty of Sport and Physical Education University of Novi Sad (No-47-10-09/2021/1). The patients/participants provided their written informed consent to participate in this study.

## Author contributions

JO and MJ wrote the manuscript, revised the manuscript, overviewed previous studies, and discussed the results. NR and DC performed the analysis. All authors contributed to the article and approved the submitted version.

## Funding

The data used in this study were collected within the research project called Physical activity and effects of dynamic neuromuscular stabilization on functional mobility during COVID-19 pandemic (Register number: 142-451-2273/2021-01/02), which was conducted by the Faculty of Sport and Physical Education, University of Novi Sad, and financed by the Provincial Secretariat for Higher Education and Scientific research.

## Conflict of interest

The authors declare that the research was conducted in the absence of any commercial or financial relationships that could be construed as a potential conflict of interest.

## Publisher's note

All claims expressed in this article are solely those of the authors and do not necessarily represent those of their affiliated organizations, or those of the publisher, the editors and the reviewers. Any product that may be evaluated in this article, or claim that may be made by its manufacturer, is not guaranteed or endorsed by the publisher.
